# Fibrin Clot Properties in Aneurysmal Coronary Artery Disease: CARE-ANEURYSM Study

**DOI:** 10.3390/ijms27209059

**Published:** 2026-10-13

**Authors:** Jakub Chmiel, Magdalena Kopytek, Maciej Dąbrowski, Sylwia Iwańczyk, Karol Musiał, Patrycja Woźniak, Julianna Chmiel, Łukasz Partyka, Michał Ząbczyk, Joanna Natorska, Piotr Musiałek

**Affiliations:** 1 Department of Cardiac and Vascular Diseases, Institute of Cardiology, Jagiellonian University Medical College, 31-202 Krakow, Poland; 2 Krakow Center for Medical Research and Technologies, St. John Paul II Hospital, 31-202 Krakow, Poland; 3 St. John Paul II Hospital, 31-202 Krakow, Poland; 4 Doctoral School of Medical and Health Sciences, Jagiellonian University Medical College, 31-530 Krakow, Poland; 5 Department of Thromboembolic Disorders, Institute of Cardiology, Jagiellonian University Medical College, 31-202 Krakow, Poland; 6 Department of Interventional Cardiology and Angiology, National Institute of Cardiology, 04-628 Warsaw, Poland; 7 1st Department of Cardiology, Poznan University of Medical Sciences, 61-848 Poznan, Poland; 8 KCRI Angiographic Core Laboratory, 30-347 Krakow, Poland

**Keywords:** coronary artery aneurysm, coronary artery ectasia, fibrin clot, oxidative stress

## Abstract

Coronary artery ectasias and aneurysms (CAEAs) are present in up to 5% of patients undergoing coronary angiography. Recent studies indicated an independent association between CAEA and the risk of myocardial infarction but the underlying mechanisms have not yet been elucidated. We evaluated the relationship between CAEA and fibrin clot properties in 60 consecutive CAEA subjects and 60 clinically/angiographically matched controls enrolled across three tertiary centers. Fibrin clot permeability coefficient (K_s_), clot lysis time (CLT), and levels of 3-nitrotyrosine, an oxidative stress marker, were assessed. Among patients with CAEA, 52 (86.7%) were male, the median age was 66.0 (59.5–74.1) years, and 30 (50.0%) had concomitant coronary artery disease. Compared with controls, CAEA subjects had 14% lower K_s_ (*p* = 0.036), 25% longer CLT (*p* = 0.001), and 21% higher 3-nitrotyrosine levels (*p* = 0.002). Multivariable regression analysis showed an association of CAEA with lower K_s_, longer CLT, and higher 3-nitrotyrosine levels (all *p* < 0.05). In CAEA patients, 3-nitrotyrosine levels correlated with K_s_ (r = −0.31, *p* = 0.016) and CLT (r = 0.27, *p* = 0.037). With increasing CAEA peak-to-reference diameter ratio, K_s_ decreased (*p*-trend = 0.017), and 3-nitrotyrosine levels increased (*p*-trend = 0.002). In conclusion, we found that CAEA is independently associated with unfavorably altered fibrin clot properties, reflected by denser clot structure and reduced susceptibility to fibrinolysis. Our findings suggest that oxidative stress may contribute to unfavorable modification of fibrin clot properties in CAEA. With an increase in CAEA size, there is an increase in oxidative stress and worsening of the fibrin clot phenotype (CARE-ANEURYSM, NCT05183373).

## 1. Introduction

Coronary artery ectasias and aneurysms (CAEAs) are present in up to 5% of patients undergoing invasive coronary angiography [1]. The pathogenesis of CAEA is not fully understood [2]. The frequent coexistence of CAEA and coronary artery disease (CAD) suggests overlapping pathophysiological mechanisms; however, CAEA also develops in the absence of CAD [1]. Impaired prognosis in CAEA has traditionally been attributed to concomitant CAD but recent studies suggest that CAEA may be independently associated with future cardiovascular events such as recurrence of myocardial infarction (MI) [3,4]. The mechanisms underlying this increased cardiovascular risk in patients with CAEA remain unknown.

The fibrin clot phenotype reflects the structural and functional properties of plasma fibrin clots [5]. Prothrombotic features include dense, poorly permeable fibrin networks (reduced clot permeability, K_s_) and resistance to fibrinolysis (prolonged clot lysis time, CLT). This prothrombotic fibrin clot phenotype has been associated with future thromboembolic events in patients with cardiovascular disease and it might contribute to the increased thrombotic risk in patients with CAEA [5,6]. Our pilot work, assessing 10 patients, suggested a low K_s_ in CAEA [7]. However, cardiovascular risk factors are common in patients with CAEA, and it remains unknown whether the suggested unfavorable alteration of the fibrin clot phenotype is related to CAEA itself or to the accompanying cardiovascular risk factor burden [1,7]. Recently, enhanced oxidative stress has been implicated in CAEA [8,9], and there is evidence that increased oxidative stress can promote unfavorable modification of fibrin clot properties [10,11].

We evaluated the hypothesis that, compared with matched controls, patients with CAEA display a more prothrombotic fibrin clot phenotype reflected by reduced K_s_ and prolonged CLT, and that oxidative stress may contribute to these alterations. The present multicenter study extends our pilot observations [7] through comparison with clinically and angiographically matched controls in a large cohort and by examining the relationships of fibrin clot properties with oxidative stress and the angiographic extent of CAEA.

## 2. Results

### 2.1. Patient Characteristics

Among 60 patients with CAEA, 52 (86.7%) were male, the median age was 66.0 (59.5–74.1) years, and 30 (50.0%) had concomitant CAD. Demographic and clinical characteristics by study group are presented in Table 1. Patients with CAEA had lower high-density lipoprotein cholesterol (1.2 vs. 1.3 mmol/L, *p* = 0.002) levels and higher hematocrit (43.7% vs. 42.1%, *p* = 0.019). No other differences were found between the CAEA patients and controls.

### 2.2. Angiographic Characteristics

**Figure 1:**
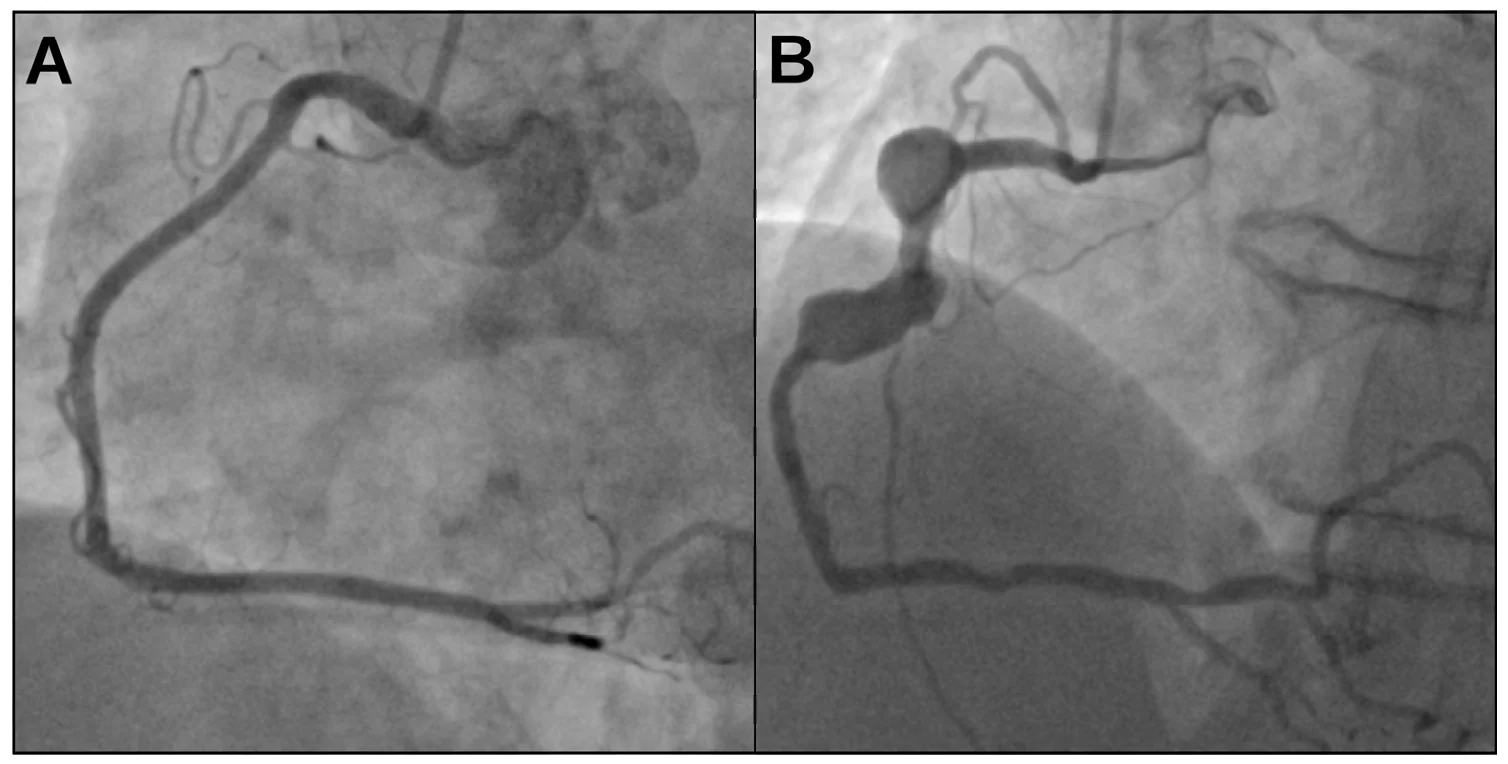
**Coronary angiograms of a control patient and a patient with CAEA.** Representative angiographic examples from a matched control without CAEA (**A**) and a patient with CAEA in the right coronary artery (**B**). CAEA, coronary artery ectasia and aneurysm.

A total of 108 CAEA lesions were identified in 60 patients. Twenty-nine (48.3%) patients had one CAEA, 18 (30%) had two, nine (15%) had three, and four (6.7%) had four CAEA. The right coronary artery was the most commonly affected vessel (n = 46, 42.6%; example in Figure 1), followed by the left anterior descending artery and the circumflex artery (both n = 31, 28.7%). The median CAEA peak-to-reference diameter ratio was 1.92 (1.70–2.16), and the median peak CAEA diameter was 5.76 (5.07–6.79) mm.

### 2.3. Clotting and Oxidative Stress

**Figure 2:**
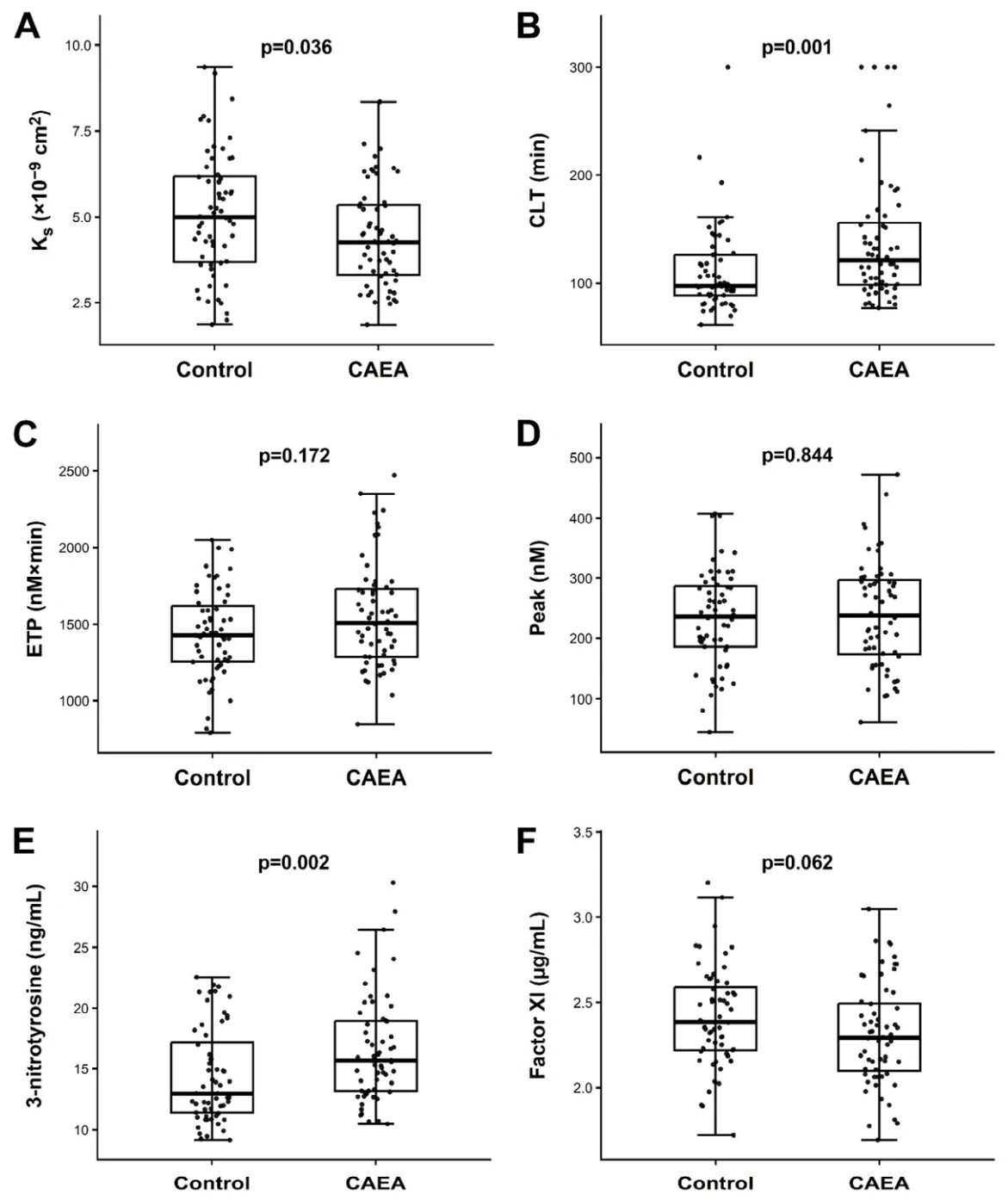
**Clotting and oxidative stress parameters in matched controls and patients with CAEA.** (**A**) Fibrin clot permeability coefficient (K_s_); (**B**) clot lysis time (CLT); (**C**) endogenous thrombin potential (ETP); (**D**) peak thrombin concentration; (**E**) 3-nitrotyrosine concentration; and (**F**) factor XI concentration. Patients with CAEA had lower K_s_, longer CLT, and higher 3-nitrotyrosine levels, compared with matched controls without CAEA. They also tended to have lower Factor XI levels. *p*-Values are for between-group comparisons using the Wilcoxon rank-sum test. Boxes show medians and interquartile ranges; whiskers extend to 1.5 × the interquartile range, and dots represent individual patients. CAEA, coronary artery ectasia and aneurysm.

**Figure 3:**
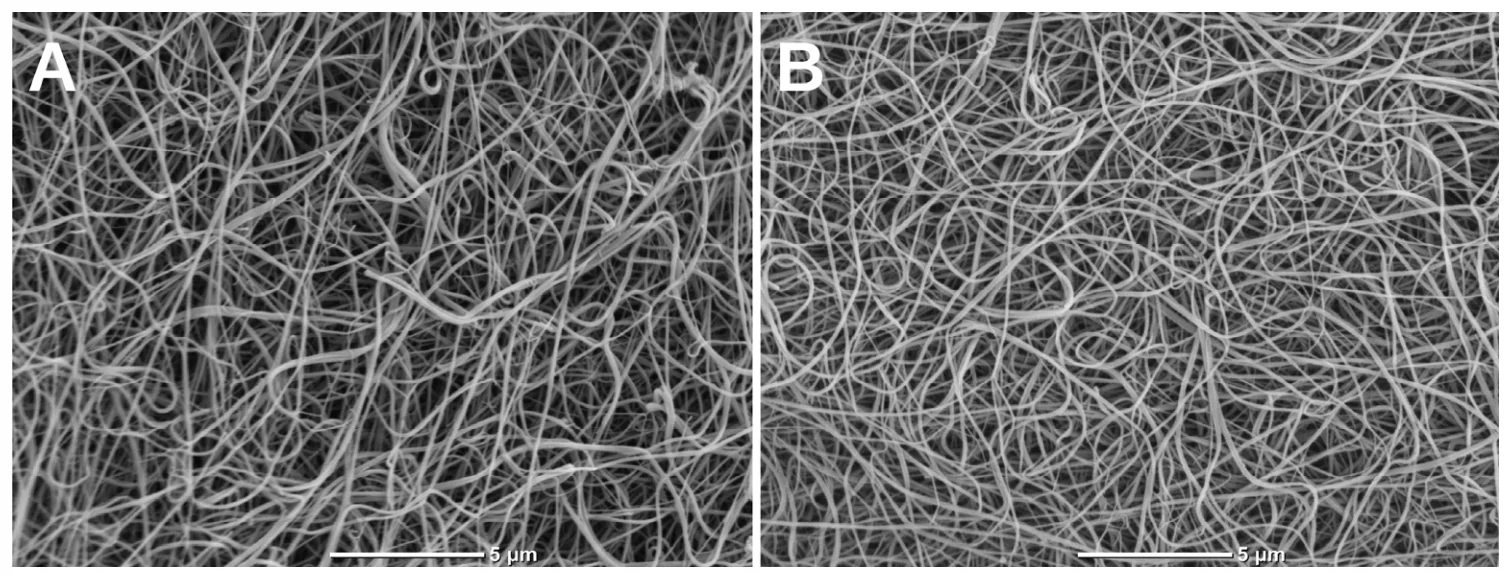
**Fibrin clot architecture in a control patient and a patient with CAEA.** Illustrative scanning electron microscope images of fibrin clots from a control patient (**A**) and a patient with CAEA (**B**). Magnification, ×5000; scale bars, 5 μm. CAEA, coronary artery ectasia and aneurysm.

Compared with controls, patients with CAEA had 11% lower fibrinogen levels (*p* = 0.022) but D-dimer levels did not differ (Table 2). Patients with CAEA had 14% lower K_s_ (*p* = 0.036) and 25% longer CLT (*p* = 0.001) than matched controls (Figure 2, Table 2). In a subset of 10 patients with CAEA and 10 controls, quantitative analysis of scanning electron microscopy images showed smaller fibrin network pores in patients with CAEA than in controls (0.516 ± 0.178 vs. 1.023 ± 0.192 μm, *p* < 0.001). Illustrative images are shown in Figure 3 (further examples are provided in Figure S1). 3-nitrotyrosine levels were 21% higher in the CAEA group than in controls (*p* = 0.002, Table 2). No differences were observed for endogenous thrombin potential (ETP) or peak thrombin (all *p* > 0.05, Table 2). Factor XI (FXI) levels tended to be lower in CAEA patients (*p* = 0.062, Table 2). After adjustment for sex, age, CAD, diabetes mellitus, and atrial fibrillation, CAEA remained associated with lower fibrinogen (*p* = 0.021) and higher 3-nitrotyrosine levels (*p* = 0.006, Table 3). After additional adjustment for fibrinogen, CAEA also remained associated with lower K_s_ (*p* = 0.006) and longer CLT (*p* = 0.002, Table 3). Both associations were also significant without adjustment for fibrinogen (K_s_: B = −0.7, 95% CI −1.3 to −0.1, *p* = 0.025; CLT: B = 27.9 min, 95% CI 10.4 to 45.4, *p* = 0.002). CAEA was not associated with D-dimer levels, FXI, or ETP (all *p* > 0.05, Table 3). Sensitivity analyses supported the conclusions of the primary analyses (Tables S1 and S2). In patients with CAEA, 3-nitrotyrosine levels correlated with K_s_ (r = −0.31, *p* = 0.016) and CLT (r = 0.27, *p* = 0.037); no such correlations were observed in controls (both *p* > 0.05; Figure S2).

### 2.4. Angiographic Extent of CAEA

**Figure 4:**
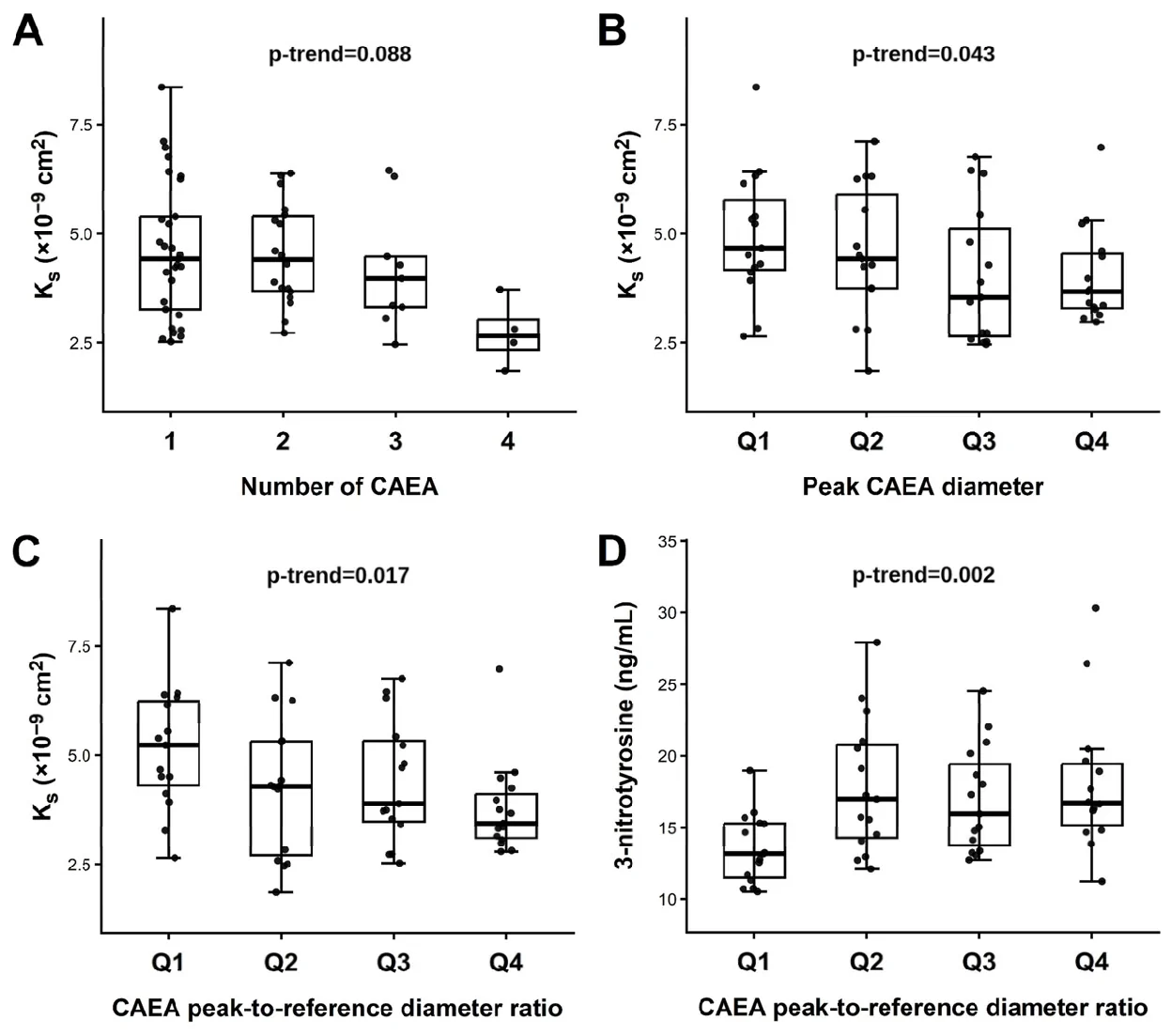
**Angiographic extent of CAEA and prothrombotic profile.** (**A**) Fibrin clot permeability coefficient (K_s_) according to the number of CAEA; (**B**) K_s_ according to peak CAEA diameter; (**C**) K_s_ according to the CAEA peak-to-reference diameter ratio; and (**D**) 3-nitrotyrosine concentration according to the CAEA peak-to-reference diameter ratio. In patients with CAEA, K_s_ decreased with increasing CAEA size. Furthermore, 3-nitrotyrosine levels increased with increasing CAEA size. *p*-Trend values were assessed using two-sided Jonckheere–Terpstra tests with 100,000 permutations. Boxes show medians and interquartile ranges; whiskers extend to 1.5 × the interquartile range, and dots represent individual patients. CAEA, coronary artery ectasia and aneurysm; Q, quartile.

K_s_ decreased across increasing quartiles of maximum CAEA diameter (*p*-trend = 0.043) and maximum peak-to-reference diameter ratio (*p*-trend = 0.017), whereas the trend across lesion-number categories was not statistically significant (*p*-trend = 0.088; Figure 4). 3-nitrotyrosine levels increased across quartiles of maximum peak-to-reference diameter ratio (*p*-trend = 0.002; Figure 4). No significant trends were observed for CLT in relation to CAEA angiographic extent (all *p*-trend > 0.05).

### 2.5. CAEA in the Presence and Absence of Concomitant CAD

In exploratory comparisons among patients with CAEA, those with CAD had a higher prevalence of prior acute coronary syndrome (ACS; *p* = 0.004); otherwise, no differences in clinical characteristics, including medication use, were observed between the groups. Patients with CAEA in the absence of CAD had 17% higher ETP (*p* = 0.025) than patients with CAEA and concomitant CAD (Table S3).

## 3. Discussion

This multicenter study substantially extends our pilot observations [7] by now evaluating a large cohort of patients with CAEA that were clinically and angiographically matched with controls, and by examining the relationships of fibrin clot properties with oxidative stress and angiographic extent of CAEA. The main findings are the following: (1) CAEA is independently associated with a prothrombotic fibrin clot phenotype, reflected by denser clot structure and reduced susceptibility to fibrinolysis; (2) higher 3-nitrotyrosine levels correlate with lower K_s_ and longer CLT in patients with CAEA, indicating an association between oxidative stress and unfavorable fibrin clot alterations in CAEA; and (3) with increasing CAEA size, there is a reduction in K_s_ and an increase in 3-nitrotyrosine levels, suggesting a worsening of the unfavorable fibrin clot phenotype and an increase in oxidative stress with increasing CAEA size.

For a long time, CAEA had been considered an incidental angiographic finding, with adverse prognosis attributed mainly to concomitant CAD [12]. Today, there is an increasing body of data suggesting that CAEA may be per se associated with adverse cardiovascular outcomes [3,4]. Evidence is particularly compelling in patients with MI, in whom CAEA is independently associated with cardiac death (hazard ratio 7.97) and subsequent MI (hazard ratios up to 3.90) [3,4]. Stent thrombosis has been proposed as a potential leading mechanism of MI recurrence [4], suggesting an increased propensity to thrombus formation and/or a thrombus phenotype more resistant to endogenous fibrinolysis. Indeed, patients with infarct-related CAEA undergoing primary percutaneous coronary intervention show greater thrombus burden, impaired myocardial reperfusion, and more frequent “no-reflow” phenomenon and distal embolization [13,14]. Coronary computed tomography angiography identifies intra-CAEA thrombus in a majority of CAEA patients [15].

Our pilot work suggested low K_s_ values in subjects with CAEA [7]. Current findings importantly extend these preliminary data by showing that CAEA is independently associated with unfavorably modified fibrin clot properties (Table 3). Moreover, we presently found that CAEA is associated with increased plasma 3-nitrotyrosine levels, a marker of oxidative stress (Table 3), and that among patients with CAEA 3-nitrotyrosine levels correlate negatively with K_s_ and positively with CLT (Figure S2). These findings are consistent with prior evidence that fibrinogen nitration may promote an unfavorable fibrin clot phenotype [10]. Nitration of tyrosine residues within the fibrinogen *β*-chain may enhance lateral aggregation of fibrin fibers and has been linked to faster fibrin formation, increased clot stiffness, and reduced susceptibility to fibrinolysis [10]. Our findings suggest that the prothrombotic fibrin clot phenotype in CAEA is not explained by increased fibrinogen concentration or thrombin generation (Table 3) or concomitant CAD (Table S3). The correlations of K_s_ and CLT with 3-nitrotyrosine support the hypothesis that oxidative modifications, including fibrinogen nitration, may contribute to the observed clot phenotype; however, fibrinogen nitration was not measured directly and no mediation analysis was performed. Notably, we found a progressive increase in oxidative stress accompanied by a simultaneous decrease in K_s_ as CAEA size increased (Figure 4). We hypothesize that oxidative stress may link CAEA size [8,9] with an unfavorable fibrin clot phenotype through a potential mechanistic pathway including fibrinogen nitration [10,11]; this might potentially contribute to the thrombotic processes implicated in adverse clinical outcomes in CAEA [3,4,16,17,18,19]. This hypothesis requires further mechanistic investigation. A larger CAEA size may be associated with a higher risk of cardiac events in patients with Kawasaki disease; however, whether CAEA size is related to thromboembolic risk in adults remains unknown [20]. Previously, a stepwise size-dependent worsening of fibrin clot properties has been reported in abdominal aortic aneurysms—from controls to patients with small abdominal aortic aneurysms and the large ones [21].

Optimal pharmacological treatment in patients with CAEA remains undetermined. Evidence regarding antithrombotic therapy in patients with CAEA is limited, and most available data are derived from retrospective studies suggesting a lower risk of ischemic events in patients with CAEA receiving antithrombotic therapy [3,22]. The present study does not provide evidence regarding the efficacy or clinical benefit of anticoagulation in patients with CAEA. The single exploratory randomized trial to date was small and limited to 12 months of follow-up [23]. In patients with CAEA and prior ACS, the primary composite clinical endpoint occurred numerically less frequently in those on clopidogrel plus rivaroxaban than in those managed on aspirin plus clopidogrel (1/30 vs. 4/32 patients) but the difference did not reach statistical significance [23]. Our observations highlight the need for appropriately powered prospective randomized studies with assessment of fibrin clot phenotype at baseline and long follow-up to determine whether a prothrombotic fibrin clot phenotype is associated with an increased risk of thromboembolic events in patients with CAEA and whether the clot phenotype can guide antithrombotic management.

## 4. Limitations

This study has several limitations. First, our findings support an association between CAEA and an unfavorable fibrin clot phenotype, but they do not establish that CAEA causes a prothrombotic state, that oxidative stress mediates this association, or that the observed fibrin clot phenotype translates into an increased risk of clinical thrombotic events. However, the associations observed in the present study provide a rationale for further mechanistic and prospective studies. Second, the study included patients who had undergone invasive coronary angiography; therefore, the findings may not be generalizable to all patients with CAEA. However, coronary angiography remains the standard method for the diagnosis and assessment of CAEA. Third, 3-nitrotyrosine was used as a systemic marker of oxidative stress, but direct assessment of fibrinogen nitration or other oxidative modifications of fibrinogen was not performed. Fibrinogen is particularly susceptible to oxidative post-translational modifications and has been reported to be approximately 20-fold more vulnerable to oxidation than albumin [11]. Despite adjustment for prespecified covariates, residual confounding by unmeasured factors, including plasminogen activator inhibitor-1 (PAI-1) and lipoprotein(a), remains possible [5,24]. *p*-Values were not adjusted for multiple comparisons; therefore, nominally significant findings, particularly those from secondary and exploratory analyses, should be interpreted with caution. Finally, the study was not designed to evaluate clinical outcomes; therefore, whether unfavorably altered fibrin clot properties identify patients with CAEA at increased risk of future thrombotic events requires prospective validation.

## 5. Materials and Methods

### 5.1. Study Population

The Inflammation and Clotting Abnormalities in AneuRysmal Coronary Artery DiseasE (CARE-ANEURYSM) study is a prospective, multicenter observational study. Between February 2022 and October 2025, we enrolled a total of 120 adults who had undergone invasive coronary angiography within the preceding 12 months: 60 patients with CAEA and 60 matched controls without CAEA from three tertiary cardiovascular centers (St. John Paul II Hospital, Krakow, Poland; National Institute of Cardiology, Warsaw, Poland; University Hospital, Poznan, Poland). In the CAEA group, 30 consecutive patients with CAEA in the absence of CAD and 30 consecutive patients with CAEA and concomitant CAD were recruited. The control group was composed of patients without CAEA who underwent coronary angiography during the study period and it was frequency-matched to patients with CAEA based on demographic and clinical characteristics. The key exclusion criteria were ACS within the previous 6 months, current dual antiplatelet therapy, New York Heart Association class IV heart failure, known malignancy, and known inherited thrombophilia. Demographic characteristics and medical history were collected; laboratory data were processed centrally. CAEA was defined as a coronary artery dilatation exceeding the reference segment diameter by at least 50% [1]. For each CAEA lesion, the reference diameter was defined as the mean of the adjacent non-dilated proximal and distal segment diameters [25,26]. Qualitative and quantitative analyses of deidentified coronary angiograms were conducted by two blinded investigators—an interventional cardiologist and a core laboratory analyst working together, using dedicated software (CAAS Workstation 8.5, Pie Medical Imaging, Maastricht, The Netherlands) in accordance with the angiographic core laboratory standard operating procedures. For each CAEA lesion, peak CAEA diameter and CAEA peak-to-reference diameter ratio were measured. The maximum value of each measure was taken to describe the greatest (absolute and relative) dilatation. CAD was defined as ≥50% luminal diameter stenosis in at least one major epicardial coronary artery on invasive coronary angiography.

This study was approved by the Bioethics Committee of the Jagiellonian University (1072.6120.154.2021; applicable to the three study centers). Written informed consent was obtained from all patients prior to enrollment, and the study was conducted in accordance with the Declaration of Helsinki. The CARE-ANEURYSM study was registered with ClinicalTrials.gov (NCT05183373).

### 5.2. Laboratory Investigations

Fasting venous blood samples were collected between 8:00 and 10:00 a.m. Blood drawn into citrated tubes was centrifuged at 2000× *g* at 20 °C for 20 min. Blood drawn into serum tubes was centrifuged at 1600× *g* at 4 °C for 10 min. Plasma and serum aliquots were stored at −80 °C until batch analysis. For participants receiving a direct oral anticoagulant (*n* = 17 in the CAEA group and *n* = 14 in the control group), blood was collected at the expected trough time point: 24–28 h after the last dose of rivaroxaban and 12–18 h after the last dose of apixaban or dabigatran. Plasma samples from these participants were treated in vitro with DOAC-Stop (Haematex Research, Sydney, Australia) to minimize potential interference from residual anticoagulant activity. Common laboratory parameters, including lipid and liver profiles, blood cell counts, glucose, creatinine, and C-reactive protein, were assayed by routine laboratory techniques. D-dimer concentrations were reported in fibrinogen equivalent units. Fibrinogen was determined using the Clauss method.

### 5.3. Fibrin Clot Analysis

Fibrin clot permeability was determined as previously described [27]. Briefly, citrated plasma was mixed with 20 mM calcium chloride and 1 U/mL human thrombin (Sigma-Aldrich, St. Louis, MO, USA). The clot-containing tube was connected to a Tris buffer reservoir (0.05 mol/L Tris-HCl, 0.15 mol/L NaCl, pH 7.5), and the volume flowing through the gels was measured over 1 h. The K_s_ was calculated from the equation: K_s_ = Q × L × η/(t × A × Δp), where Q is the volume of buffer during percolation time (t), L is the length of the fibrin gel, η is the viscosity of the liquid, A is the cross-sectional area (cm^2^), and Δp is the differential pressure (dyne/cm^2^). K_s_ reflects the pore size within the fibrin network; lower K_s_ values indicate a denser, less permeable fibrin network [28]. The inter-assay coefficient of variation was <7%. In addition, scanning electron microscopy images of fibrin clots were obtained from the first 10 consecutive patients with CAEA and the first 10 consecutive controls. Mean pore size was calculated by averaging measurements of the pore in the center of each of 20 fields in each of three images per patient; measurements were performed jointly by two blinded analysts. Pore size was quantified using ImageJ software (version 1.50i, National Institutes of Health, Bethesda, MD, USA) [29].

CLT was used to determine fibrinolytic capacity [30]. Briefly, citrated plasma was mixed with 15 mM calcium chloride, 0.5 U/mL human thrombin (Merck KGaA, Darmstadt, Germany), and 15 μM phospholipid vesicles (Rossix, Mölndal, Sweden), and then supplemented with 18 ng/mL recombinant tissue-type plasminogen activator (Boehringer Ingelheim, Ingelheim am Rhein, Germany). The mixture was transferred to a microtiter plate, and absorbance was measured at 405 nm at 37 °C. CLT was defined as the time between the midpoint of the clear-to-maximum-turbid transition and the midpoint of the maximum-turbid-to-clear transition. The intra- and inter-assay coefficients of variation were 6%.

### 5.4. Endogenous Thrombin Potential (ETP)

Calibrated Automated Thrombography (Thrombinoscope BV, Maastricht, The Netherlands) was used to measure ETP. The fluorometer (Ascent Reader, Thermo Labsystems Oy, Helsinki, Finland) was used to generate the area under the curve at 37 °C. Briefly, 20 μL of tissue factor-based activator (PPP Reagent containing 5 pmol/L recombinant tissue factor and 4 μmol/L phospholipid vesicles; Diagnostica Stago, Asnières-sur-Seine, France) and FluCa solution (Diagnostica Stago, Asnières-sur-Seine, France) were added to 80 μL of platelet-poor plasma. The area under the curve of thrombin formed during the recording time represented the ETP, while the maximum concentration of thrombin generated was reported as peak thrombin.

### 5.5. ELISA Tests

Plasma 3-nitrotyrosine and FXI concentrations were measured using the ELISA kits (both Thermo Fisher Scientific, Waltham, MA, USA), according to the manufacturer’s instructions.

### 5.6. Statistical Analysis

The prespecified primary analyses compared K_s_, CLT, and 3-nitrotyrosine between patients with CAEA and controls, with CLT used for the sample size calculation. Prespecified secondary analyses included fibrinogen, D-dimer, FXI, ETP, peak thrombin, and associations with CAEA angiographic extent. Prespecified supportive analyses examined correlations of 3-nitrotyrosine with K_s_ and CLT. Comparisons according to CAD status within the CAEA group were exploratory. The study was designed to provide at least 90% power to detect a 10% between-group difference in CLT, using a two-sided alpha level of 0.05. Assuming a standard deviation equal to 16% of the mean CLT [31], 55 patients were required per group. The target sample size was set at 60 patients per group; the estimated power was 92.4%.

Continuous data are presented as mean ± standard deviation or median (interquartile range), and categorical data as absolute frequencies and percentages. Differences between the CAEA and control groups were assessed using the chi-square test or Fisher’s exact test, as appropriate, for categorical variables and the Wilcoxon rank-sum test for continuous variables. Between-group differences in mean pore size derived from scanning electron microscopy images were assessed using the Wilcoxon rank-sum test. For each outcome, multivariable linear regression was used with CAEA entered as a binary exposure (CAEA = 1, control = 0) and the outcome modeled in its original units. Results are presented as unstandardized B coefficients with 95% confidence intervals (CI), representing adjusted CAEA–control differences. Sex, age, CAD, diabetes mellitus, and atrial fibrillation were selected a priori based on clinical relevance and published associations with fibrin clot properties [5,31]. The adjustment set was restricted to limit model complexity in a cohort of 120 participants. Models for K_s_ and CLT additionally included fibrinogen concentration as a well-known determinant of clot properties [5]. Regression diagnostics and sensitivity analyses are described in the Supplementary Materials. Correlations were assessed using Spearman correlation coefficients. Within the CAEA group, trends across lesion-number categories (1–4) and rank-based quartiles of maximum CAEA diameter and maximum peak-to-reference diameter ratio were assessed using two-sided Jonckheere–Terpstra tests with 100,000 permutations. Among the clotting and oxidative stress biomarkers, missing data were confined to D-dimer (3/120), leaving 117 complete cases; all other regression models included 120 participants. Other missing data were limited to descriptive laboratory variables, including total cholesterol (*n* = 2) and high-density lipoprotein cholesterol, non-high-density lipoprotein cholesterol, triglycerides, alanine aminotransferase, and aspartate aminotransferase (*n* = 4 each). No data imputation was performed. All tests were two-sided. *p*-Values were not adjusted for multiple comparisons, and *p* < 0.05 was interpreted as statistically significant. Statistical analysis was performed using R software (version 4.5.2; R Foundation for Statistical Computing, Vienna, Austria).

## 6. Conclusions

We found that CAEA is independently associated with a prothrombotic fibrin clot phenotype characterized by denser clot structure and reduced susceptibility to fibrinolysis. Enhanced oxidative stress in CAEA may contribute to the observed alterations. Further large-scale studies are warranted to determine whether fibrin clot properties identify patients with CAEA at increased thrombotic risk and whether anticoagulation improves clinical outcomes.

## Figures and Tables

**Table 1:** Patient characteristics across study groups.

Variable	Control (*n* = 60)	CAEA (*n* = 60)	*p*-Value
Male, *n* (%)	52 (86.7%)	52 (86.7%)	1.000
Age, years	68.4 (57.4–75.2)	66.0 (59.5–74.1)	0.553
**Cardiovascular risk factors**			
CAD, *n* (%)	30 (50.0%)	30 (50.0%)	1.000
Hyperlipidemia, *n* (%)	60 (100.0%)	60 (100.0%)	1.000
Hypertension, *n* (%)	49 (81.7%)	51 (85.0%)	0.624
Diabetes mellitus, *n* (%)	19 (31.7%)	20 (33.3%)	0.845
Atrial fibrillation, *n* (%)	13 (21.7%)	13 (21.7%)	1.000
CKD, *n* (%)	10 (16.7%)	10 (16.7%)	1.000
Current smoker, *n* (%)	7 (11.7%)	6 (10.0%)	0.769
Obesity, *n* (%)	26 (43.3%)	25 (41.7%)	0.853
**Medical history**			
Prior ACS, *n* (%)	18 (30.0%)	27 (45.0%)	0.090
Prior stroke, *n* (%)	7 (11.7%)	3 (5.0%)	0.186
Prior PE, *n* (%)	1 (1.7%)	1 (1.7%)	1.000
**Laboratory parameters and medications**			
eGFR, mL/min/1.73 m^2^	75 (66–89)	82 (69–93)	0.176
Total cholesterol, mmol/L	3.8 (3.1–4.8)	3.6 (2.7–4.2)	0.062
LDL-C, mmol/L	2.0 (1.4–2.9)	1.7 (1.4–2.6)	0.259
HDL-C, mmol/L	1.3 (1.2–1.5)	1.2 (0.9–1.4)	**0.002**
Non-HDL-C, mmol/L	2.3 (1.9–3.5)	2.3 (1.7–3.0)	0.339
Triglycerides, mmol/L	1.1 (0.9–1.4)	1.3 (1.0–2.0)	0.190
ALT, U/L	24 (16–43)	25 (19–40)	0.420
AST, U/L	24 (18–30)	23 (20–31)	0.523
CRP, mg/L	1.3 (0.9–2.7)	1.5 (0.7–3.1)	0.900
WBC, ×10^9^/L	7.0 (6.1–8.1)	7.0 (5.8–9.1)	0.591
Hemoglobin, g/dL	14.2 (13.3–15.1)	14.8 (13.8–15.8)	0.062
Hematocrit, %	42.1 (39.2–44.1)	43.7 (41.4–46.1)	**0.019**
Platelet count, ×10^9^/L	213 (167–255)	200 (163–245)	0.429
Aspirin, *n* (%)	45 (75.0%)	44 (73.3%)	0.835
Statin, *n* (%)	52 (86.7%)	56 (93.3%)	0.224

Data are presented as median (interquartile range) or *n* (%). Bold values indicate statistical significance (*p *< 0.05). Missing data: total cholesterol, *n* = 2; HDL-C, non-HDL-C, triglycerides, ALT, and AST, *n* = 4 each. ACS, acute coronary syndrome; ALT, alanine aminotransferase; AST, aspartate aminotransferase; CAD, coronary artery disease; CAEA, coronary artery ectasia and aneurysm; CKD, chronic kidney disease; CRP, C-reactive protein; eGFR, estimated glomerular filtration rate; HDL-C, high-density lipoprotein cholesterol; LDL-C, low-density lipoprotein cholesterol; non-HDL-C, non-high-density lipoprotein cholesterol; PE, pulmonary embolism; WBC, white blood cell count.

**Table 2:** Clotting and oxidative stress parameters across study groups.

Variable	Control *n* = 60	CAEA *n* = 60	*p*-Value
Fibrinogen, g/L	3.8 (3.3–4.2)	3.4 (3.0–3.9)	**0.022**
D-dimer, µg/L	444 (244–1218)	430 (271–724)	0.821
Factor XI, µg/mL	2.4 (2.2–2.6)	2.3 (2.1–2.5)	0.062
3-nitrotyrosine, ng/mL	13.0 (11.4–17.2)	15.7 (13.2–19.0)	**0.002**
ETP, nM × min	1430 (1257–1642)	1510 (1287–1730)	0.172
Peak thrombin, nM	238 (186–290)	238 (174–297)	0.844
K_s_, ×10^−9^ cm^2^	5.0 (3.7–6.2)	4.3 (3.3–5.3)	**0.036**
CLT, min	97 (89–126)	121 (99–156)	**0.001**

Data are presented as median (interquartile range). Bold values indicate statistical significance (*p *< 0.05). D-dimer: *n* = 58 in the control group and *n* = 59 in the CAEA group; all other parameters: *n* = 60 per group. CAEA, coronary artery ectasia and aneurysm; CLT, clot lysis time; ETP, endogenous thrombin potential; K_s_, fibrin clot permeability coefficient.

**Table 3:** Adjusted association between CAEA and clotting and oxidative stress parameters.

Variable	CAEA, B (95% CI)	*p*-Value
Fibrinogen, g/L	−0.4 (−0.7 to −0.1)	**0.021**
D-dimer, μg/L	−142 (−424 to 139)	0.324
Factor XI, μg/mL	−0.1 (−0.2 to 0.0)	0.068
3-nitrotyrosine, ng/mL	2.8 (0.9 to 4.7)	**0.006**
ETP, nM × min	78 (−63 to 219)	0.282
Peak thrombin, nM	−2 (−37 to 33)	0.907
K_s_, ×10^−9^ cm^2^	−0.8 (−1.4 to −0.25)	**0.006**
CLT, min	29.5 (11.5 to 47.4)	**0.002**

D-dimer: *n* = 117; all other models: *n* = 120. Adjustment included male sex, age, CAD, diabetes mellitus, atrial fibrillation; models for K_s_ and CLT were additionally adjusted for fibrinogen level. Bold values indicate statistical significance (*p *< 0.05). B, unstandardized regression coefficient; CAD, coronary artery disease; CAEA, coronary artery ectasia and aneurysm; CI, confidence interval; CLT, clot lysis time; ETP, endogenous thrombin potential; K_s_, fibrin clot permeability coefficient.

## Data Availability

The data presented in this study are available on request from the corresponding author. The data are not publicly available due to ethical restrictions.

## Supplementary Materials

The following supporting information can be downloaded at https://www.mdpi.com/article/10.3390/ijms27209059/s1.
