# Supplementary material for: Fibrin Clot Properties in Aneurysmal Coronary Artery Disease: CARE-ANEURYSM Study
Source: Int J Mol Sci. 2026 Oct 13;27(20):9059. doi: 10.3390/ijms27209059 (PMC13705159; doi:10.3390/ijms27209059)
Supplement: Supplementary file 1 [file ijms-27-09059-s001.zip › ijms-4581800-supplementary.pdf]

Fibrin Clot Properties in Aneurysmal Coronary Artery Disease:
CARE-ANEURYSM Study

Chmiel J et al.

Supplementary Materials

Table of contents:

Supplementary Methods 2
Supplementary Results..3
Table S1. Studentized bootstrap sensitivity analyses of the adjusted associations between CAEA and clotting and oxidative stress parameters 4
Table S2. Influence diagnostics and leave-one-out sensitivity analyses of the adjusted models..........5
Table S3. Clotting and oxidative stress parameters among patients with CAEA with and without concomitant CAD 6
Figure S1. Further examples of fibrin clot architecture in controls and patients with CAEA ....7.
Figure S2. Correlations between 3-nitrotyrosine levels and fibrin clot properties in patients with CAEA and controls 8

Supplementary Methods

Regression diagnostics

Linearity was assessed using component-plus-residual plots for continuous covariates. Residual-versus-fitted plots and the Koenker–Breusch–Pagan test were used to assess homoscedasticity, and Q–Q plots and the Shapiro–Wilk test were used to examine residual distributions. Potentially influential observations were identified using Cook's distance $>4/n$, where n denotes the number of participants included in the corresponding model. No observations were excluded solely on the basis of influence diagnostics.

Sensitivity analyses

To assess the robustness of the estimated CAEA associations, studentized bootstrap 95% confidence intervals were calculated during revision using 20,000 resamples of complete participant records drawn with replacement separately within the CAEA and control groups. Resampling preserved the original group sizes and the relationships between outcomes and covariates. Each resampled dataset was analyzed using the same model specification as the original dataset. Conventional ordinary least-squares standard errors were used for studentization in the original and resampled models. Confidence limits were obtained by inverting the empirical 2.5th and 97.5th percentiles of the studentized bootstrap statistic.

Leave-one-out analyses were performed by sequentially omitting each participant with complete data for a given model and refitting the same model, irrespective of the participant's Cook's distance. The ranges of the CAEA coefficients and corresponding two-sided ordinary least-squares P values were recorded.

Supplementary Results

Linear regression diagnostics

Regression diagnostics did not identify clear evidence of nonlinearity or heteroscedasticity. Residual distributions departed from normality in the models for fibrinogen, D-dimer, 3-nitrotyrosine, ETP, peak thrombin, and CLT. Cook's distance >4/n flagged 31 unique participants as potentially influential in at least one model.

Sensitivity analyses

Studentized bootstrap confidence intervals supported the original conclusions (Table S1). The associations of CAEA with lower fibrinogen, higher 3-nitrotyrosine, lower K_s, and longer CLT retained their direction and nominal statistical significance in every leave-one-out refit. For FXI and ETP, P values fell below 0.05 in some refits, although neither association was statistically significant in the full-sample model (Table S2).

Table S1. Studentized bootstrap sensitivity analyses of the adjusted associations between CAEA and clotting and oxidative stress parameters

Variable	n	B	Bootstrap-t 95% CI
Fibrinogen, g/L	120	-0.4	-0.7 to -0.1
D-dimer, µg/L	117	-142	-461 to 121
Factor XI, µg/mL	120	-0.1	-0.21 to 0.01
3-nitrotyrosine, ng/mL	120	2.8	1.1 to 5.2
ETP, nM × min	120	77.7	-96.5 to 206.6
Peak thrombin, nM	120	-2.1	-42.2 to 30.4
K_s, $\times 10^{-9}$ cm^2	120	-0.8	-1.4 to -0.2
CLT, min	120	29.5	12.2 to 48.4

B represents the ordinary least-squares adjusted CAEA–control difference in the original outcome units, with CAEA coded as 1 and controls as 0. All models included sex, age, coronary artery disease, diabetes mellitus, and atrial fibrillation; K_s and CLT models additionally included fibrinogen concentration. CAEA, coronary artery ectasia and aneurysm; CI, confidence interval; CLT, clot lysis time; ETP, endogenous thrombin potential; K_s, fibrin clot permeability coefficient.

Table S2. Influence diagnostics and leave-one-out sensitivity analyses of the adjusted models

Outcome	n	Cook's D >4/n, n	B	Leave-one-out B range	Leave-one-out p range
Fibrinogen, g/L	120	11	−0.4	−0.4 to −0.3	0.009 to 0.034
D-dimer, µg/L	117	5	−142	−192 to −58	0.161 to 0.624
Factor XI, µg/mL	120	6	−0.1	−0.1 to −0.09	0.034 to 0.104
3-nitrotyrosine, ng/mL	120	1	2.8	2.1 to 3.0	0.003 to 0.009
ETP, nM × min	120	3	77.7	63.7 to 127.1	0.020 to 0.373
Peak thrombin, nM	120	4	−2.1	−5.7 to 9.0	0.528 to 0.997
K_s, $\times 10^{-9}$ cm^2	120	4	−0.8	−1.0 to −0.7	0.001 to 0.013
CLT, min	120	6	29.5	27.0 to 32.8	<0.001 to 0.004

B represents the adjusted CAEA vs control difference in the original outcome units, with CAEA coded as 1 and controls as 0. All models included sex, age, coronary artery disease, diabetes mellitus, and atrial fibrillation; K_s and CLT models additionally included fibrinogen concentration. CAEA, coronary artery ectasia and aneurysm; CLT, clot lysis time; ETP, endogenous thrombin potential; K_s, fibrin clot permeability coefficient.

Table S3. Clotting and oxidative stress parameters among patients with CAEA with and without concomitant CAD

Variable	CAEA without CAD n=30	CAEA with CAD n=30	p-value
Fibrinogen, g/L	3.3 (3.0-3.9)	3.5 (3.1-3.9)	0.647
D-dimer, µg/L	473 (272-603)	415 (231-790)	0.876
Factor XI, µg/mL	2.3 (2.1-2.5)	2.3 (2.1-2.5)	0.756
3-nitrotyrosine, ng/mL	15.5 (12.9-18.5)	16.0 (14.2-19.1)	0.395
ETP, nM×min	1676 (1308-1909)	1428 (1289-1570)	**0.025**
Peak thrombin, nM	276 (177-314)	226 (161-285)	0.115
K_s, $\times 10^{-9}$ cm^2	4.4 (3.7-6.2)	3.9 (2.9-4.8)	0.099
CLT, min	126 (98-160)	117 (99-153)	0.824

Data are presented as median (interquartile range). D-dimer was available in 30 patients without CAD and 29 patients with CAD. CAD, coronary artery disease; CAEA, coronary artery ectasia and aneurysm; CLT, clot lysis time; ETP, endogenous thrombin potential; K_s, fibrin clot permeability coefficient.

Figure S1. Further examples of fibrin clot architecture in controls and patients with CAEA.

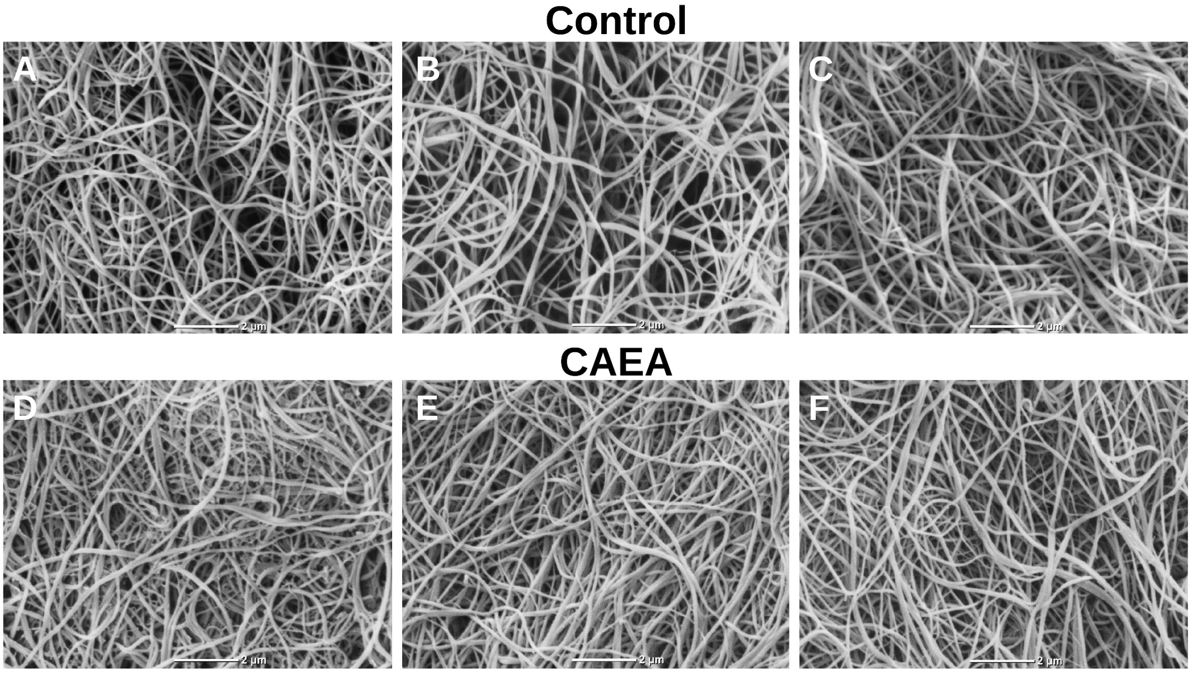

Illustrative scanning electron microscopy images of fibrin clots from controls (A–C) and patients with CAEA (D–F). Magnification, ×10,000; scale bars, 2 μm. CAEA, coronary artery ectasia and aneurysm.

Figure S2. Correlations between 3-nitrotyrosine levels and fibrin clot properties in patients with CAEA and controls.

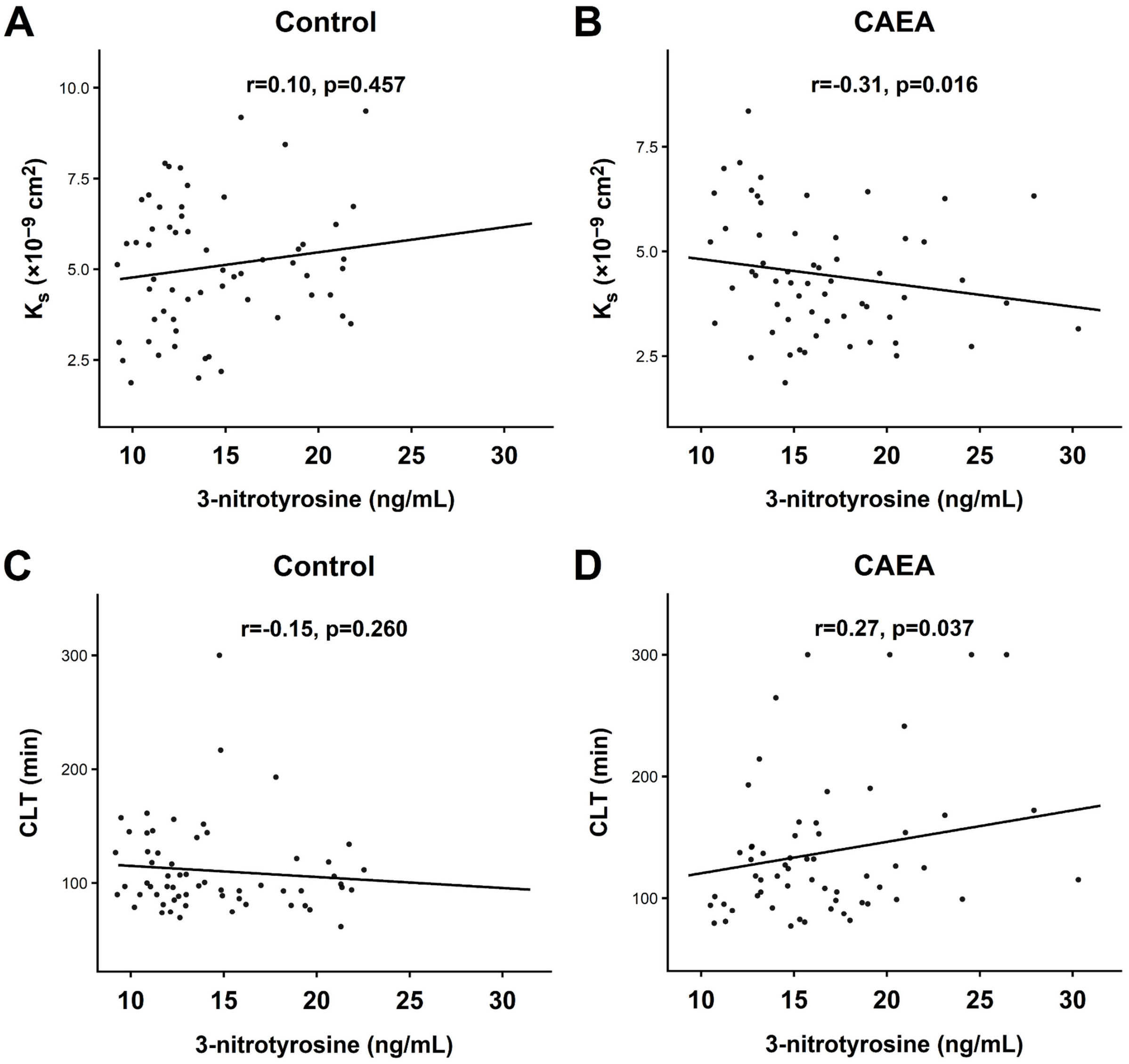

In patients with CAEA, 3-nitrotyrosine levels correlated negatively with K_s and positively with CLT, whereas neither correlation was statistically significant in controls. Spearman correlation coefficients (r) and p values are shown. Lines represent linear regression fits. CAEA, coronary artery ectasia and aneurysm; CLT, clot lysis time; K_s, fibrin clot permeability coefficient.
